# Assessing the Impact of Video-Based Assignments on Health Professions Students’ Social Presence on Web: Case Study

**DOI:** 10.2196/11390

**Published:** 2018-11-26

**Authors:** Jennie C De Gagne, Sang S Kim, Ellen R Schoen, Hyeyoung K Park

**Affiliations:** 1 School of Nursing Duke University Durham, NC United States; 2 Red-Cross College of Nursing Chung-Ang University Seoul Republic of Korea; 3 Vanderbilt University Medical Center Nashville, TN United States

**Keywords:** case study, engagement, multimedia, Web-based learning, social presence, students’ experience

## Abstract

**Background:**

Web-based education is one of the leading learning pedagogies in health professions education. Students have access to a multitude of opinions, knowledge, and resources on Web, but communication among students in Web-based courses is complicated. Technology adds a filter that makes it difficult to decipher the emotions behind words or read nonverbal cues. This is a concern because students benefit more from Web-based classes when they have a high perception of social presence. To enhance social presence on Web, we planned to use video-based assignments (VBAs) that encourage students to interact with each other.

**Objective:**

This case study examines the impact of VBAs on health professions students and their experiences with the technology. This study aims to provide information to the growing body of literature about strategies to develop social presence on Web.

**Methods:**

A total of 88 students from various nursing programs participated in the study. While the control group comprised 36 students who submitted only written-based assignments (WBAs), the experimental group of 52 students submitted VBAs besides WBAs. No enrolled student had previously participated in the course, and there were no repeaters in either of the groups. Both groups participated in a weekly survey comprising 4 open-ended questions and 3 Likert items on a scale of 1-5 (1=strongly disagree and 5=strongly agree). The social presence questionnaire assessed by the experimental group comprised 16 items and a 5-point Likert scale in which higher scores represented higher levels of social presence. While quantitative data were analyzed using descriptive statistics, qualitative responses were analyzed using content analysis.

**Results:**

No significant differences were noted between the groups regarding the program (F_1,87_=0.36, *P*=.54). Regarding students’ engagement, no statistically significant difference was observed between the 2 groups (t_14_=0.96, *P*=.35). However, the experimental group’s average score for engagement was slightly higher (4.29 [SD 0.11]) than that of the control group (4.21 [SD 0.14]). Comparison of the total number of responses to the weekly engagement survey revealed 88.0% (287/326) as either strongly agree or agree in the control group, whereas 93.1% (525/564) in the experimental group. No statistically significant difference was observed between VBAs and WBAs weeks (t_6_=1.40, *P*=.21) in the experimental group. Most students reported a positive experience using VBAs, but technical issues were barriers to embracing this new approach to learning.

**Conclusions:**

This study reveals that social presence and engagement are positively associated with student learning and satisfaction in Web-based courses. Suggestions are offered to enhance social presence on Web that could generate better learning outcomes and students’ experiences.

## Introduction

Distance education is a phenomenon where students are presented course information remotely, with or without interaction with their instructors and fellow students. Although this may seem like a recent development, distance education appeared as early as 1880 when students received courses through the mail or radio. However, it was not until the 1990s that we saw the modern form of distance education through the development of the internet and other information technologies. Today, Web-based education—the acquisition of knowledge and skills through a mediated Web-based infrastructure—is one of the leading learning pedagogies [1]. More than 6.7 million students in the United States engage in Web-based learning, and an increase in the number of Web-based courses is a key component in the growth plans of many colleges and universities [2]. Proponents of electronic learning applaud its ability to reach a multitude of students, thus increasing the diversity and perspectives in the internet-based classroom. Students do not have to meet in a set place or at a set time, allowing for increased flexibility in accessing information. This flexibility is especially important to the growing number of nontraditional learners who may be balancing the responsibilities of full-time jobs and parenthood with their educations [3].

While students in Web-based courses have access to a multitude of opinions, knowledge, and resources, they rarely, if ever, have face-to-face interactions with their professors or classmates. This is a concern because learners get the greatest benefit from Web-based courses when they can have meaningful interactions with their classmates or establish their social presence [4]. The term *social presence* was coined by Short et al to describe the feeling of talking to a real person in mediated communication such as Web-based communication [5]. A sense of community naturally evolves in a traditional classroom setting where students spend time together and engage in face-to-face conversations. There, students can consider what is said and in what tone and read their peers’ nonverbal cues during discussions. However, communication among students in Web-based courses is more complicated because technology adds a filter that makes it difficult to decipher the emotions behind words [6]. Indeed, students in Web-based courses cite feelings of isolation and a lack of connection with classmates as key factors that hindered their success in Web-based learning [1]. Undoubtedly, these feelings can contribute to dropout rates for Web-based courses, which are 10%-20% higher than traditional courses [7,8]. The necessary use of mediated communication in Web-based courses and subsequent feelings of isolation in Web-based learning may be why students in Web-based courses feel less social presence than their face-to-face counterparts.

The social presence is a critical factor for students’ engagement and satisfaction and has a clear impact on student outcomes such as deeper learning or greater cognitive absorption [9]. For example, students who had higher perceptions of social presence also had better-perceived learning outcomes [4]. Similarly, Horzum demonstrated that social presence has a positive association with student satisfaction in Web-based courses [10]. Therefore, students are likely to benefit more from Web-based education if they can develop social presence and interact with other learners.

One salient strategy that can enhance social presence and learners’ engagement is to use technology, such as video-based assignments (VBAs), to allow active student interactions [11]. With the wide availability of desktop and laptop cameras, students can take advantage of audio and visual expression to present information in original ways. Assignments with multimedia deliverables can encourage engagement with course materials while creating a sense of community as students share the video production process. Furthermore, when students have the capability of personalizing instructional materials themselves and have control over their own learning and production, they exhibit higher levels of satisfaction and motivation [12]. This case study aims to examine the impact of VBAs on students and other means of enhancing students’ engagement and social presence in a Web-based class. Furthermore, students’ perceptions and experiences with educational technology are reported.

## Methods

### Case Study: Description of the Course

The data used in this study came from graduate-level health professions students who took the course, *Facilitating Student Learning in Academic Setting*. This 2-credit, 8-week course focuses on theories and principles that provide a foundation for the diverse learning needs of adults and strategies to meet these needs. Major topics of this student-centered course include the principles of adult learning, the concepts of learning styles, domains of learning, and innovative teaching strategies. At the end of the course, students are expected to be able to (1) analyze how context, motivation, generational differences, and other factors affect students’ engagement in the learning process; (2) propose appropriate teaching practices to facilitate learning in the 3 domains of learning; (3) examine the evidence that underlies teaching practices to identify gaps that need to be addressed through scholarly efforts; and (4) formulate well-written learning objectives for each domain of learning. The number of students enrolled in this course ranges from semester to semester, generally between 10 and 35. Regardless of the class size, students work in small groups of 4-5 so that their course loads are unlikely to be affected by the number of students enrolled.

Sakai, an electronic learning management system (LMS), is an open-source learning environment that provides instructors with tools to support their teaching. Powered by this LMS, the course first exposes students to learning materials on the class website, then follows these lessons with weekly activities such as problem-solving discussions. Sakai orientation is not provided at the course level but the school level, as this LMS is used throughout the university. Students work in groups of 4-5 to facilitate collaborations and engagement. The course does not have a textbook, as no single book covers the entire course, and textbooks often contain only basic information, which quickly becomes outdated. Thus, materials used in this course are current, and technology resources (eg, help desk and hardware loan program) are readily available to students. Basic system requirements for the course are a desktop or laptop computer, internet access, up-to-date virus protection software, a webcam, and a headset with microphone. All learning materials, activities, and evaluations are in Sakai in a logical sequence, making the entire course an effective and engaging experience.

#### Innovation of the Course

Historically, this course has been text-centric (ie, written discussion forums and written assignments); driven by faculty’s commitment to creating a more active, engaging learning environment, in the Fall 2015 semester, that changed. The course became multimedia-oriented with the introduction of 2 new Sakai tools as follows: (1) streaming media (WarpWire, a video-capture/sharing platform with plug-in support for different LMSs); and (2) peer assessment for students’ assignments. The midterm and final course evaluations were used to determine the success of these 2 new tools; they indicated that students’ presentations through WarpWire and peer assessment were effective in facilitating connectivity and presence on Web. However, WarpWire and peer-assessment tools had limited capacity and took a considerable amount to troubleshoot. An alternative technology, the YouSeeU software (YouSeeU, Loveland, CO, USA) was chosen, as it offered functions unavailable in WarpWire and the peer-assessment tool. In addition, it enabled students to create video presentations synchronized to a PowerPoint slide deck and participate in multimedia group projects either synchronously or asynchronously. They could also provide peer assessment by annotating and synchronizing comments on videos. Students were provided with specific rubrics for each assignment. A university grant purchased 60 YouSeeU licenses to provide the VBA and peer-assessment functions.

#### Implementation

In this study, 52 students in the experimental group were enrolled in Spring 2017 (n=27), Fall 2017 (n=11), and Spring 2018 (n=14). These students were given VBAs in addition to written-based assignments (WBAs). The control group (n=36) with WBAs consisted of students enrolled in the Fall 2016 semester. Students in both the control and experimental groups completed weekly engagement surveys. This survey included 4 open-ended questions with 3 items using a 5-point Likert scale indicating the degree of agreement with each statement (eg, 1=strongly disagree to 5=strongly agree; see Multimedia Appendix 1). These items were derived from a modified Brookfield’s Critical Incident Questionnaire designed to help instructors understand their students’ learning experiences better [13].

All students in the experimental group who completed the VBAs in weeks 1, 4, and 8 were encouraged to share their experiences with these assignments and assess their social presence perception by responding to a voluntary questionnaire. This questionnaire was based on the social presence scale for Web-based learning environments developed by Kilic et al [14] and the social presence questionnaire designed by Lin [15]. The social presence questionnaire used in this case study consisted of 16 items with a 5-point Likert scale for which higher scores represented higher levels of social presence. The internal consistency of the items was acceptable, with a Cronbach alpha of .752 and .782 when excluding the reverse-worded item, Question 3 (Q3): *I hesitated to ask questions to others during Web-based discussions*.

The purpose and process of each assignment were explained during the course overview at the beginning of each semester. In addition, students were informed of the course evaluation survey by *Announcement* in Sakai and assured that the anonymously recorded responses would be used for course improvement purposes only. The Institutional Review Board of Duke University deemed this course improvement project to be exempt.

### Evaluation

The quantitative survey data were organized and analyzed using Excel and SPSS 23.0 (SPSS Inc, Chicago, IL). Students’ characteristics were assessed using descriptive statistics, and between-group homogeneity was tested by an *F* test. In addition, differences in variables between 2 groups were evaluated using a *t* test. Qualitative responses were analyzed to identify principal messages in the content. A recursive review of the transcriptions was conducted among authors by consensus decision making to achieve full agreement with the findings [16].

## Results

### Principal Results

A total of 88 students participated in this study, consisting of 36 students in the control group and 52 students in the experimental group. No significant differences were noted between the groups with respect to the program in which students were enrolled. In the control group, 50% (18/36), 47% (17/36), and 3% (1/36) of the students were enrolled in the master’s in nursing (MSN) program, doctor of nursing practice (DNP)/philosophy of doctorate in nursing (PhD), and others, respectively. In the experimental group, 40% (21/52) were enrolled in the MSN program, and 60% (31/52) in the DNP/PhD program.

### Levels of Weekly Engagement

No statistically significant difference was observed between the 2 groups (*t*_14_=0.96, *P*=.35) in terms of students’ engagement. However, the average score of students’ engagement was higher in the experimental group (4.29 [SD 0.11]) than that in the control group (4.21 [SD 0.14]). Comparing the total number of engagement responses, 88.0% (287/326) were reported as either *strongly agree* or *agree* to the responses in the control group, while 93.1% (525/564) of the responses were reported in the experimental group. No statistically significant difference was noted between WBA and VBA weeks in the experimental group (*t*_6_=1.40, *P*=.21; Tables 1 and 2).

**Table 1:** Students’ engagement between 2 groups.

Groups	Total number of responses (N)	Responses to weekly engagement surveys, n (%)					Course			
		Strongly agree	Agree	Undecided	Disagree	Strongly disagree	Weekly engagement, mean (SD)		*t*	*P*
Control group (n=36)	326	133 (40.8)	154 (47.2)	21 (6.4)	10 (3.1)	8 (2.5)	4.21 (0.14)		0.964	.35
Experimental group (n=52)	564	211 (37.4)	314 (55.7)	31 (5.5)	8 (1.4)	0	4.29 (0.11)			

**Table 2:** Students’ engagement between written-based assignment and video-based assignment weeks.

Weeks	Total number of responses (N)	Responses to weekly engagement surveys, n (%)						Course		
		Strongly agree	Agree	Undecided	Disagree	Strongly disagree		Weekly engagement, mean (SD)	*t*	*P*
Written-based assignment weeks (2, 3, 5, 6, and 7)	339	126 (37.2)	201 (59.3)	9 (2.7)	3 (0.9)	0		4.22 (0.09)	1.400	.21
Video-based assignment weeks (1, 4, and 8)	225	85 (37.8)	113 (50.2)	22 (9.8)	5 (2.2)	0		4.33 (0.11)		

### Perceptions of Social Presence

**Figure 1:**
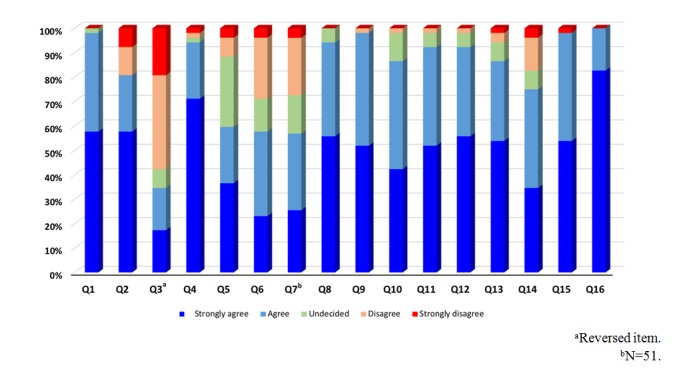
Social presence in the YouSeeU group (N=52).

The experimental group that completed both WBAs and VBAs had a mean total score of 4.22 (SD 0.40) on the social presence survey. The item with the highest mean score was Q16: *Overall, I felt respected and valued by the Web-based* *learning community members* (4.83 [SD 0.38]). Students’ responses indicated that they believed they had clearly expressed their ideas to their peers during Web-based discussions (4.79 [SD 0.41]), felt like they were a member of a community during Web-based discussions (4.60 [SD 0.80]), shared their opinions whether they agreed or disagreed during discussions (4.56 [SD 0.54]), and felt comfortable during YouSeeU activities (4.50 [SD 0.61]). The item with the lowest mean score was Q6: *The group project helped me accomplish the assignment with higher quality than if I were working alone* (3.48 [SD 1.21]; Figure 1; Table 3).

**Table 3:** Social presence in the YouSeeU group (N=52).

Question	Items	Mean (SD)
Q1	I shared my opinions whether I agreed or disagreed during Web-based discussions.	4.56 (0.54)
Q2	I clearly expressed my ideas to other students during Web-based discussions.	4.79 (0.41)
Q3^a^	I hesitated to ask questions to others during Web-based discussions.	3.63 (1.33)
Q4	I felt like I was a member of a group during Web-based discussions.	4.60 (0.80)
Q5	I felt that verbal discussion forums were more engaging than written Web-based discussion forums.	3.52 (1.08)
Q6	The group project helped me accomplish the assignment with higher quality than if I were working alone.	3.48 (1.21)
Q7^b^	Without the YouSeeU platform, it would have been difficult to complete a group assignment.	3.51 (1.22)
Q8	I felt comfortable expressing my feelings during YouSeeU activities.	4.50 (0.61)
Q9	I shared my opinions whether I agreed or disagreed during YouSeeU activities.	4.48 (0.61)
Q10	I felt closer to other students during YouSeeU activities than during the written Web-based discussion forums.	4.27 (0.74)
Q11	I clearly expressed my ideas to other students during YouSeeU activities.	4.42 (0.70)
Q12	I felt I came to know the other students better during YouSeeU activities than during the written Web-based discussion forums.	4.46 (0.70)
Q13	The use of YouSeeU platform has helped me to complete oral presentation assignments.	4.33 (0.92)
Q14	Overall, I feel that YouSeeU platform was intuitive and easy to use.	3.88 (1.15)
Q15	Overall, I feel that YouSeeU platform provided me with unique Web-based learning experiences.	4.48 (0.70)
Q16	Overall, I felt respected and valued by the Web-based learning community members.	4.83 (0.38)

^a^Reversed item.

^b^N=51.

### Qualitative Results

Most students in both groups stated that their engagement in discussion forums by responding to other students’ posts, sharing experiences, and applying new knowledge to their teaching practices was positive. The instructor gave the prompt, but discussions were facilitated by both students and the instructor. Some of the comments were as follows:

I enjoyed searching the readings on my own volition and picking the readings of my interest and using these readings to support my findings in response to form prompts and responding to other classmates’ forums.

Articulating my thoughts and supporting them by research really pulled everything together for me to allow a deeper understanding of the content.

Most engagement was felt when having exchanges with my peers in terms of stories and experiences.

I feel really passionate about the content we are learning, and I get excited when I can make connections with my past and current experiences.

In addition to these common engagement points, the YouSeeU group students stated that using the new technology was challenging but also refreshing and motivating. They said:

It was my first time utilizing the YouSeeU program. I had used [A] and [B] in the past but only during live interactive Web-based sessions. This app is unique (and really neat). It allows someone to post and upload recordings for later viewing by faculty and other students.

I enjoy the video forums. Watching people’s faces and body language helps to drive the points home for me (more than the written word).

The YouSeeU platform always makes me feel more engaged and connected with the class.

Some students said that they felt least engaged when listening to recorded lectures, writing discussion posts, and spending time with the new technology. Furthermore, they felt less engaged when they were not prepared to respond to peer posts and had insufficient time to reflect on others’ responses.

I felt most distant just before posting the video. The fear of the unknown. Would I get the video right? Would I answer appropriately? Would the posting upload? I did feel a bit anxious just before, but my angst was not necessary.

I did have technical difficulties, which frustrated me when I posted on YouSeeU.

It’s a personal thing really. When I log in and see the great work of others, I immediately feel behind the curve and out of the loop. It’s not the course. It’s me, my generation, and my learning style.

## Discussion

### Principal Findings

This case study examined multimedia-facilitated assignments and their impact on students’ engagement and social presence in the Web-based classroom. No statistically significant differences were observed in the level of engagement between the 2 groups, but students in the experimental group found VBAs to be effective and acceptable learning strategies. The experimental group reported a high level of social presence. Although this study did not measure the retention of knowledge directly, the level of cognitive absorption might have been influenced by this enhanced social presence in the experimental group [8]. While most students reported positive experiences using VBAs, technical issues posed a barrier to embracing this new approach to learning. This is consistent with other studies that found nursing students and nurse educators often struggling with emerging technologies [17,18]. Even though YouSeeU has a sync slides function for group work, the group oral presentation that required synchronization was reported as one of the most challenging tasks, as group members had to coordinate their different time zones and work schedules.

Pedagogy in the student-to-course interaction is an important aspect in developing social presence. Valenzuela et al hypothesized, “pedagogical approaches may be more important than technology in determining the effectiveness of [Web-based] courses.” [19]. In addition, the use of asynchronous communication in Web-based learning can lead to feelings of discomfort among students [20]. Students who are not familiar with Web-based education and what is expected of learners in the Web-based environment have a difficult time establishing a clear identity in virtual classrooms [20]. These feelings of discomfort or failure in establishing an identity may hinder social presence in the Web-based learning environment.

Furthermore, the social presence can be affected by students’ preconceived attitudes toward Web-based communications [21]. To that end, attempts were made to develop innovative ways to increase students’ interaction and social presence. For example, Wang and Chen incorporated a group presentation and a virtual reality simulation into a course that required cross-cultural communication and the utilization of new technologies [22]. In their study, class discussion on group presentations had a 26% higher response rate than discussions on class readings, and 80% of students felt the group projects improved their communication skills. In another study by Akcaoglu and Lee, creating smaller groups of 4-5 students for discussions led to a higher level of social presence in terms of sociability, social space, and group cohesion [23]. These authors concluded that manipulating group size could positively affect students’ relationship building in asynchronous class discussions.

This study adopted VBAs to facilitate students’ engagement and enhance their social presence. This case study indicates that in both experimental and control groups, students felt comfortable voicing their opinions in the discussion forums. This is may be because most of them had taken Web-based courses before and knew how to share opinions through Web. As current or future educators in health professions, students were also highly receptive to Web-based communication tools. They believed that these tools were useful in developing their presentation skills and preparing them for their teaching careers. The learner-driven activities in VBAs, such as creating video clips and getting feedback from peers, made students feel as though learning was a partnership rather than a competition. Finally, students were encouraged to take a voluntary engagement survey every week, which may have underscored the importance of engagement in Web-based learning.

### Limitations and Future Directions

The main limitation of this study is that the assessment of students’ engagement and social presence was based on self-reporting rather than observation of behavior or mediating indicators. Self-reporting is effective in investigating students’ satisfaction with a course, perceived engagement, and social presence, but its validity and accuracy are limited [24]. Utilizing self-reporting, Gunawardena and Zittle were among the first researchers to measure social presence in Web-based education with their social presence scale and student satisfaction scale [25]. These scales are widely used together to determine the degree of correlation and the mediating effect of social presence on students’ satisfaction [4,10]. In contrast to the self-reporting style of the social presence survey, observing patterns of students’ communication in Web-based forums was developed [26]. In this instrument, the social presence density of students’ communication within the forum is calculated and classified as affective, cohesive, or interactive [26]. This case study did not show a significant difference between students with WBAs only and those with both WBAs and VBAs. If both self-reporting and observation had been used to collect data, results might have been different.

Another limitation of this study is that the instructor’s perspective was not a part of the study outcomes. Bolliger et al incorporated the social presence principles of faculty-to-student interaction and student-to-student interaction to develop a faculty satisfaction survey for Web-based courses; this survey can identify key areas for faculty and institutional improvement in developing social presence and increasing students’ satisfaction in the Web-based environment [27]. It is important to measure the faculty presence, as instructors who create a high-level presence may potentially influence students’ attitudes and behaviors, making it difficult for them to share their perspectives through Web [28]. Despite these limitations, this case study provides an important contribution to the growing body of literature supporting the use of active and engaging learning strategies for developing social presence on Web.

As trends indicate a growing demand for Web-based education, it is essential that health professions stay in front of this rapidly developing field. There are a number of ways for faculty and institutions to enhance students’ engagement and social presence in Web-based learning. Foremost, faculty participation is likely to facilitate social presence. Their clear communication on the course design and organization will improve students’ engagement. Faculties that transition from a face-to-face to a Web-based classroom note that students on Web need more instructions about their assignments than students in a traditional classroom setting [2]. Paul and Cochran suggested a comprehensive orientation for students on Web and faculty that allows students to ask questions about the LMS [3]. This orientation could also provide students with ways to increase interaction with other students and engagement with the course material. Similarly, Northcote et al developed a comprehensive training program for Web-based faculty, including on-site support, clear guidelines, and benchmarks for Web-based learning [29]. Professional development activities for Web-based faculty markedly increased their self-efficacy and eased anxiety about using new strategies [29].

In addition to the faculty’s role, the interaction between faculty and their institutions was a key component in developing social presence [3]. Most of the time, institutions have oversight as to which tools are made available to faculty. Many Web-based faculty members are limited by the support provided to them within Web-based LMSs. Indeed, the LMS was ranked as one of the most important aspects of student learning [19], while faculty often reported dissatisfaction with institutional support for Web-based courses [27]. Similarly, faculty reported being apprehensive about their capacity to navigate the technology required to deliver a Web-based course [29]. Thus, institutional support for faculty teaching on Web can be a crucial facilitator of, or barrier to, the development of social presence in Web-based courses.

### Conclusions

This study adds to the growing body of research that social presence is a key factor in the effectiveness of Web-based education. The social presence and students’ engagement are positively associated with students’ satisfaction and are proven to improve students’ learning outcomes in Web-based courses. In addition, social presence has a positive effect on students’ cognitive absorption of the course material. The effective development of social presence in Web-based learning relies on complex interactions among students, faculty, technology, and institutions. However, there are no current “best practice” guidelines for implementing Web-based education. Much of the impetus for developing social presence in Web-based learning lies on individual faculty, institutions, and students. As these faculty members work to implement innovative strategies in Web-based learning, they must also engage in the critical revision and analysis of their Web-based courses. Strategies that reduce mediated communication, such as videoconferencing and multimedia assignments, have the potential to increase social presence in Web-based learning and provide a foundation for further course development. As Web-based education continues to become a key method of content delivery and students’ interaction, it is essential that health professions educators develop and revise Web-based pedagogy in a way that cultivates social presence in their Web-based courses.

## Appendix

Multimedia Appendix 1 Weekly Engagement Survey.

## Abbreviations

LMS: Learning Management System
VBAs: video-based assignments
WBAs: written-based assignments
