# Supplementary material for: Assessing the Impact of Video-Based Assignments on Health Professions Students’ Social Presence on Web: Case Study
Source: JMIR Med Educ. 2018 Nov 26;4(2):e11390. doi: 10.2196/11390 (PMC6288589; doi:10.2196/11390)
Supplement: Multimedia Appendix 1 [file mededu_v4i2e11390_app1.pdf]

Appendix 1: Weekly Engagement Survey

Please respond to the questions below about this week's learning activities. Your responses are anonymous.

1. I had fun in class this week.

   ☐ 5: Strongly agree
   ☐ 4: Agree
   ☐ 3: Undecided
   ☐ 2: Disagree
   ☐ 1: Strongly disagree

2. I contributed meaningfully to the discussions.

   ☐ 5: Strongly agree
   ☐ 4: Agree
   ☐ 3: Undecided
   ☐ 2: Disagree
   ☐ 1: Strongly disagree

3. I participated in the class activities most of the time this week.

   ☐ 5: Strongly agree
   ☐ 4: Agree
   ☐ 3: Undecided
   ☐ 2: Disagree
   ☐ 1: Strongly disagree

4. At what point in this week did you feel most engaged with what was happening?

5. At what point in this week did you feel most distanced from what was happening?

6. What learning activities that you took this week were affirming and helpful to you?

7. What learning activities that you took this week were puzzling or confusing to you?
